# Mechanisms and Points of Control in the Spread of Inflammation: A Mathematical Investigation

**DOI:** 10.1007/s11538-020-00709-y

**Published:** 2020-03-28

**Authors:** A. Bayani, J. L. Dunster, J. J. Crofts, M. R. Nelson

**Affiliations:** 1 Department of Physics and Mathematics, Nottingham Trent University, Clifton Campus, Nottingham, NG11 8NS UK; 2 Institute for Cardiovascular and Metabolic Research, University of Reading, Reading, RG6 6AS UK

**Keywords:** Inflammation, Resolution, Mathematical modelling, Chemotaxis, Partial differential equations

## Abstract

Understanding the mechanisms that control the body’s response to inflammation is of key importance, due to its involvement in myriad medical conditions, including cancer, arthritis, Alzheimer’s disease and asthma. While resolving inflammation has historically been considered a passive process, since the turn of the century the hunt for novel therapeutic interventions has begun to focus upon active manipulation of constituent mechanisms, particularly involving the roles of apoptosing neutrophils, phagocytosing macrophages and anti-inflammatory mediators. Moreover, there is growing interest in how inflammatory damage can spread spatially due to the motility of inflammatory mediators and immune cells. For example, impaired neutrophil chemotaxis is implicated in causing chronic inflammation under trauma and in ageing, while neutrophil migration is an attractive therapeutic target in ailments such as chronic obstructive pulmonary disease. We extend an existing homogeneous model that captures interactions between inflammatory mediators, neutrophils and macrophages to incorporate spatial behaviour. Through bifurcation analysis and numerical simulation, we show that spatially inhomogeneous outcomes can present close to the switch from bistability to guaranteed resolution in the corresponding homogeneous model. Finally, we show how aberrant spatial mechanisms can play a role in the failure of inflammation to resolve and discuss our results within the broader context of seeking novel inflammatory treatments.

## Introduction

Acute inflammation is essential to health, being the body’s response to damage, infection and foreign material; however, when dysregulated, inflammation can fail to resolve and, as such, contributes to a large variety of pathophysiological processes. While the range of pathologies that feature or arise from inflammation vary greatly (from cancer, to diabetes, to arthritis to name just a few), the cellular and chemical pathways that characterise the inflammatory response remain similar across conditions (Libby 2007). The initial inflammatory response primarily starts in the vasculature where leucocytes [neutrophils and monocytes (precursors of macrophages)] migrate into damaged tissue. Neutrophils are recruited into tissue early; these are short-lived cells that release substances that can kill bacteria but which can also be harmful to otherwise healthy tissue. Monocytes and macrophages arrive later and are phagocytes, which essentially eat foreign particles and dead or dying cells, including dead neutrophils. The balance between the cellular components of inflammation is orchestrated via a variety of pro- and anti-inflammatory mediators that, combined with the interactions between neutrophils and macrophages, control the progression of inflammation to healthy resolution or a chronic, self-perpetuating condition. Diseases characterised by chronic inflammation may be linked to the inability of acute inflammation to resolve, and specific pro-resolution pathways are now seen as alternative therapeutic targets (Fullerton and Gilroy 2016; Sugimoto et al. 2016); however, in addition, there is growing experimental evidence that the inflammatory response is characterised by spatial changes and that mechanisms such as cell motility are key in identifying how inflammatory conditions progress (Luster et al. 2005; Eming et al. 2007; Nourshargh et al. 2016; Jasper et al. 2019). There is a growing need to elucidate the mechanisms that control the interactions between the distinct cell types that drive the resolution of inflammation and, in particular, how spatial effects such as cell motility effect inflammatory outcomes, since the potential to actively manipulate these aspects of the inflammatory response exhibits great scope for development of new drugs and treatments (Libby 2007; Hunter 2012).

There are many examples of mathematical models that capture the interactions that underlie the inflammatory process, both in a generic context and tailored to particular disease scenarios, but the majority include only a single generic cell type and take a spatially averaged approach that focuses largely upon how total numbers/concentrations of cells and mediators evolve temporally in the tissue of interest. Kumar et al. (2004) proposed a model that included interactions between a generic pathogen and two classes of pro-inflammatory response that represent the combined effects of early-responding immune cells (neutrophils, mediators) and a late pro-inflammatory feedback. Having elucidated the manner in which key model parameters influence resulting outcomes, the authors suggest various therapies for persistent infectious inflammation (sepsis), with reducing the late pro-inflammatory feedback being a particular target. This work was built upon by Reynolds et al. (2006), who incorporated a time-dependent anti-inflammatory response and investigated how modulation of this response could present a route to potential new therapeutic interventions, and again by Day et al. (2010), who used a nonlinear model predictive control approach to identify therapeutic strategies.

Spatially dependent models of inflammation are comparatively sparse in existing literature, and generally lack explicit descriptions of the distinct populations of immune cells (neutrophils and macrophages) that are thought to be central to the resolution of acute inflammation. The work of Lauffenburger and Keller (1979) includes a spatial description of motile bacteria, a generic inflammatory cell type and an attractant moving in a cylindrical section of tissue that surrounds a blood vessel. This work highlights how the effectiveness of the inflammatory response depends critically upon the rates of diffusion or chemotaxis of inflammatory cells. Similarly, the work of Lauffenburger and Kennedy (1983) highlights how the chemotaxis rates can act as a switch between homogeneous and inhomogeneous steady states in a model of bacterial infection. The model of Penner et al. (2012) describes spatial interactions of a generic group of inflammatory cells, chemokines and anti-inflammatory cytokines, and exhibits interesting spatial patterns such as travelling waves, localised breathers and spatially inhomogeneous temporal oscillations. Recent advances in technology are leading to an increase in experimental data that highlight the spatial interactions underpinning inflammatory processes and mechanistic mathematical models alongside the computational techniques necessary to analyse such complex datasets are being used to interpret and elucidate the spatial interactions seen in the data (Liepe et al. 2012; Ziraldo et al. 2013; Weavers et al. 2016).

**Fig. 1:**
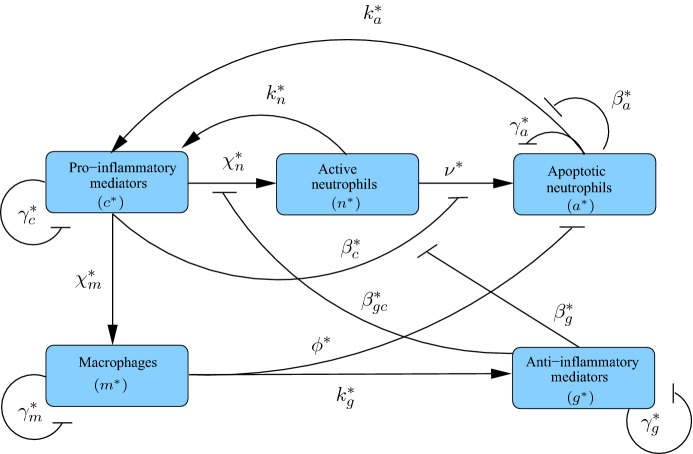
Schematic diagram illustrating the constituent interactions between populations of healthy neutrophils ($n^*$), apoptotic neutrophils ($a^*$) and macrophages ($m^*$) in response to pro- and anti-inflammatory mediators ($c^*$ and $g^*$, respectively), with associated parameters (Colour figure online)

In this article, we aim to supplement existing literature in this area by presenting a model of the acute inflammatory response that, while being generic in context, includes spatial descriptions of the distinct populations of cells thought to underlie the resolution of inflammation or progression to chronically inflamed states. By incorporating explicit descriptions of distinct, interacting cell populations, we aim to better elucidate how migration of these cells impacts upon the resolution of inflammation. We take as our starting point a previous spatially independent model (Dunster et al. 2014) depicted schematically in Fig. 1, which focuses on understanding how distinct immune cell types (neutrophils and macrophages) and pro- and anti-inflammatory mediators interact to yield either a healthy resolution of inflammation or a progression to an unhealthy self-sustained condition. We build upon this model to incorporate spatial behaviour through addition of diffusive movement of non-apoptotic cells and inflammatory mediators, plus chemotactic movement of immune cells towards areas of damage. Through numerical simulations and comparison against a bifurcation analysis of the corresponding homogeneous model, we examine how variations in key model parameters can either stimulate or eliminate spatial patterns. Finally, we reflect upon how the results of this model could inform the continuing hunt for new therapies and treatments.

## Model

In the model below we denote concentrations of generic pro- and anti-inflammatory mediators by $c^*(\mathbf {x^*},t^*)$ and $g^*(\mathbf {x^*},t^*)$, respectively, and include populations of active neutrophils, apoptotic neutrophils and macrophages (with number densities $n^*(\mathbf {x^*},t^*)$, $a^*(\mathbf {x^*},t^*)$ and $m^*(\mathbf {x^*},t^*)$, respectively). Here, $\mathbf {x^*}$ is our spatial coordinate, $t^*$ is time, and stars are used to distinguish dimensional variables from their dimensionless counterparts throughout. Our governing equations are as follows:

1a $$\begin{aligned} \frac{\partial n^*}{\partial t^*}&=-\nu ^*\displaystyle \frac{1+\frac{g^*}{\beta _g^*}}{1+\frac{c^*}{\beta _c^*}} n^*+\chi _n^*\displaystyle \frac{c^*}{1+\frac{g^*}{\beta _{gc}^*}}+D_n^*\nabla ^2n^*-\theta _n^*\nabla \cdot \left( n^*\nabla c^*\right) , \end{aligned}$$

1b $$\begin{aligned} \frac{\partial a^*}{\partial t^*}&=\nu ^*\displaystyle \frac{1+\frac{g^*}{\beta _g^*}}{1+\frac{c^*}{\beta _c^*}} n^*-\gamma _a^* a^*-\phi ^* m^* a^*,\end{aligned}$$

1c $$\begin{aligned} \frac{\partial m^*}{\partial t^*}&=\chi _m^* c^*-\gamma _m^* m^*+D_m^*\nabla ^2m^*-\theta _m^*\nabla \cdot \left( m^*\nabla c^*\right) ,\end{aligned}$$

1d $$\begin{aligned} \frac{\partial c^*}{\partial t^*}&=k_n^*\bigg (\frac{n^{*2}}{\beta _n^{*2}+n^{*2}}\bigg )+k_a^*\gamma _a^*\bigg (\frac{a^{*2}}{\beta _a^{*2}+a^{*2}}\bigg )-\gamma _c^* c^*+D_c^*\nabla ^2c^*,\end{aligned}$$

1e $$\begin{aligned} \frac{\partial g^*}{\partial t^*}&=k_g^*\phi ^*m^*a^*-\gamma _g^*g^*+D_g^*\nabla ^2g^*. \end{aligned}$$

**Fig. 2:**
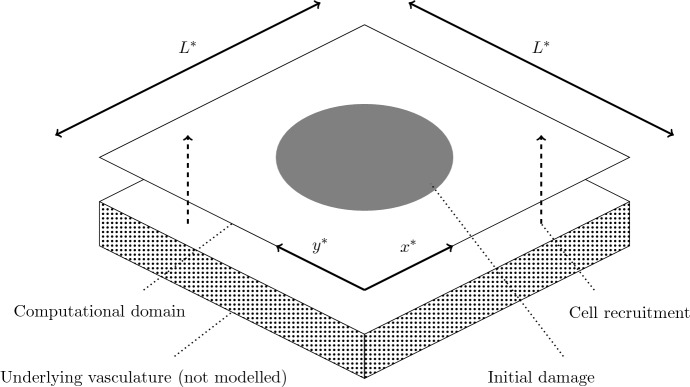
Schematic diagram of our computational domain. We solve (1) on a square domain of dimension $L^*$, subject to initial conditions that include localised damage in the centre of the domain. Dashed arrows represent recruitment of immune cells from the underlying vasculature (not modelled). Periodic boundary conditions are applied on all boundaries

As in the model of Dunster et al. (2014), our model includes recruitment of macrophages and active neutrophils in response to high levels of pro-inflammatory mediator with rates $\chi _m^*$ and $\chi _n^*$, respectively, with neutrophil recruitment also being suppressed by high levels of anti-inflammatory mediator, with associated saturation constant $\beta _{gc}^*$. Active neutrophils become apoptotic at a rate dependent upon the relative levels of pro- and anti-inflammatory mediators, as reported in previous literature (Serhan 2007; Akgul et al. 2001; Rossi et al. 2007; Lee et al. 1993) and discussed by Dunster et al. (2014); we denote the associated rate parameter and saturation constants by $\nu ^*$ and $\beta _c^*$, $\beta _g^*$, respectively. Apoptotic neutrophils are removed by macrophages of concentration $k_g^*$, causing an associated release of anti-inflammatory mediators at rate $k_g^*$, as reported in previous publications (Lawrence et al. 2002; Lawrence and Gilroy 2007; Henson 2005; Serhan and Savill 2005). Both active and apoptotic neutrophils provide saturating sources of pro-inflammatory mediator with associated parameters $k_n^*$, $k_a^*$ and saturation constants $\beta _n^*$, $\beta _a^*$, respectively. We assume that apoptotic neutrophils, macrophages and pro/anti-inflammatory mediators each decay linearly with rates $\gamma _a^*$, $\gamma _m^*$, $\gamma _c^*$, $\gamma _g^*$, respectively. In addition to the above, we develop upon the model of Dunster et al. (2014) through addition of diffusive movement of mediators and (non-apoptotic) cells and chemotactic movement of active neutrophils and macrophages up gradients of pro-inflammatory mediator concentration. We denote diffusion constants and chemotactic constants by $D_{i}^*$ and $\theta _{i}^*$, with subscripts identifying the component of interest. The above parameters are summarised in Table 1. We solve (1) on a square spatial domain of dimension $L^*$ (as shown in Fig. 2), subject to periodic boundary conditions and initial conditions that incorporate both areas of healthy tissue and tissue damage, as discussed below.

**Table 1:** Summary of the dimensional parameters appearing in (1)

Parameter	Definition
$\nu ^*$	Neutrophil apoptosis rate
$\phi ^*$	Rate of apoptotic neutrophil removal by macrophages (secondary necrosis)
$\chi _n^*$, $\chi _m^*$	Maximal rates of neutrophil/macrophage influx
$\gamma _a^*$	Rate of necrosis of apoptotic neutrophils
$\gamma _m^*$	Rate of macrophages leaving tissue
$\gamma _c^*$, $\gamma _g^*$	Rate of pro/anti-inflammatory mediator decay
$k_a^*$	Concentration of pro-inflammatory mediators produced upon apoptotic neutrophil necrosis
$k_g^*$	Concentration of anti-inflammatory mediators produced upon phagocytosis of apoptotic neutrophils by macrophages
$k_n^*$	Rate of pro-inflammatory mediator production by active neutrophils
$\beta _a^*$, $\beta _n^*$, $\beta _c^*$, $\beta _g^*$, $\beta _{gc}^*$	Saturation constants
$D_n^*$, $D_m^*$, $D_c^*$, $D_g^*$	Diffusivities of active neutrophils, macrophages and pro/anti-inflammatory mediators
$\theta _n^*$, $\theta _m^*$	Rates of neutrophil/macrophage chemotaxis

We direct the reader to Dunster et al. (2014) for further discussion of spatially independent parameters; we discuss appropriate choices for spatial parameter values in Sect. 2.1

To simplify the analysis, we rewrite (1) in terms of dimensionless variables defined as follows

2a $$\begin{aligned} n^*= & {} \frac{\chi _n^* k_a^*}{\gamma _c^*}n,\quad a^*=\frac{\chi _n^* k_a^*}{\gamma _c^*}a,\quad m^*=\frac{\chi _m^* k_a^*}{\gamma _c^*}m,\quad c^*=k_a^*c,\quad g^*=\beta _{gc}^*g, \end{aligned}$$

2b $$\begin{aligned} \mathbf {x^*}= & {} L^*\mathbf {x},\quad t^*=\frac{1}{\gamma _c^*}t, \end{aligned}$$

resulting in the following dimensionless system:

3a $$\begin{aligned} \frac{\partial n}{\partial t}&=-\nu \frac{1+\frac{g}{\beta _g}}{1+\frac{c}{\beta _c}} n+\frac{c}{1+g}+D_{n}\nabla ^2n-\theta _n\nabla \cdot \left( n\nabla c\right) , \end{aligned}$$

3b $$\begin{aligned} \frac{\partial a}{\partial t}&=\nu \frac{1+\frac{g}{\beta _g}}{1+\frac{c}{\beta _c}} n-\gamma _a a-\phi m a,\end{aligned}$$

3c $$\begin{aligned} \frac{\partial m}{\partial t}&=c-\gamma _m m+D_{m}\nabla ^2m-\theta _m\nabla \cdot \left( m\nabla c\right) ,\end{aligned}$$

3d $$\begin{aligned} \frac{\partial c}{\partial t}&=\gamma _a\frac{a^2}{\beta _a^2+a^2}+ k_n\frac{n^2}{\beta _n^2+n^2}-c+D_c\nabla ^2c,\end{aligned}$$

3e $$\begin{aligned} \frac{\partial g}{\partial t}&=k_g\phi m a-\gamma _g g+D_g\nabla ^2g, \end{aligned}$$

where dimensionless parameters are as defined in Table 2. We seek solutions to (3) on a unit square domain, subject to periodic boundary conditions.

**Table 2:** Dimensionless parameters appearing in the system (3), and their definition in terms of dimensional quantities

Spatially independent parameters			Spatial parameters		
Parameter	Expression	Baseline value	Parameter	Expression	Baseline value
$\nu$	$\nu ^*/\gamma _c^*$	0.1	$D_c$	$D_c^*/L^{*2}\gamma _c^*$	$1\times 10^{-4}$
$\phi$	$\phi ^*\chi _m^*k_a^*/\gamma _c^{*2}$	0.1	$D_g$	$D_g^*/L^{*2}\gamma _c^*$	$1\times 10^{-4}$
$\gamma _a$	$\gamma _a^*/\gamma _c^*$	1	$D_n$	$D_n^*/L^{*2}\gamma _c^*$	$1\times 10^{-5}$
$\gamma _m$	$\gamma _m^*/\gamma _c^*$	0.01	$D_m$	$D_m^*/L^{*2}\gamma _c^*$	$1\times 10^{-6}$
$\gamma _g$	$\gamma _g^*/\gamma _c^*$	1	$\theta _n$	$\theta _n^*/L^{*2}\gamma _c^*$	$1\times 10^{-5}$
$k_n$	$k_n^*/k_a^*\gamma _c^*$	0.01	$\theta _m$	$\theta _m^*/L^{*2}\gamma _c^*$	$1\times 10^{-6}$
$k_g$	$k_g^*k_a^*\chi _n^*/\beta _{gc}^*\gamma _c^*$	0.1			
$\beta _a$	$\beta _a^*\gamma _c^*/\chi _n^*k_a^*$	0.1			
$\beta _n$	$\beta _n^*\gamma _c^*/\chi _n^*k_a^*$	0.1			
$\beta _c$	$\beta _c^*/k_a^*$	0.12			
$\beta _g$	$\beta _g^*/\beta _{gc}^*$	0.01			

Also shown are baseline values used in simulations in Sect. 3. Choices of spatially independent parameter values are informed by Dunster et al. (2014); choices of spatial parameter values are discussed in Sect. 2.1

### Parameter Values

We here briefly address the question of how to obtain biologically feasible values for the dimensional parameters listed in Table 1. This is a non-trivial task since some of the required measurements are not well documented in existing literature, due to myriad reasons that include lack of clarity in some biological mechanisms (Haslett 1999; Gilroy et al. 2010), the fact that the typical short lifespan of an acute inflammatory response results in many patients presenting only after the condition has progressed beyond the acute stage, and the fact that parameter values can vary significantly between different types of tissue. We direct the reader to Dunster et al. (2014) for a thorough review of previous literature that informs our model’s spatially independent dimensional parameters, summarised in Table 1, and also their dimensionless counterparts in Table 2. Where previous literature is sufficient to provide only appropriate orders of magnitude for certain parameters, we display simulations that best illustrate the model’s full array of behaviours from a mathematical perspective.

Of key importance amongst the spatially independent parameters discussed by Dunster et al. (2014) is the rate of pro-inflammatory mediator decay, $\gamma _c^*$, since this parameter is used in the determining key timescales in our model. Existing literature reports extracellular mediator decay rates to lie between 0.7–20 per day (Waugh and Sherratt 2007; Su et al. 2009; Smith et al. 2011). Following Dunster et al. (2014), we take $\gamma _c^*=3~\text{ day }^{-1}$.

Despite measurements of spatial parameters being relatively sparse in previous literature in comparison with the vast numbers of published temporal studies of the inflammatory response, some measures of these parameters are available from both experimental studies and inferred from mathematical models. It should be noted, however, that these measurements are subject to variability across tissues. Rates of mediator diffusion reported in previous literature generally lie in the range $10^{-8}$–$10^{-6}~\text{ cm }^2\text{ s }^{-1}$—see, for example, Warrender et al. (2006), Weidemann et al. (2011), Ross and Pompano (2018) and references therein. In general, we expect the rates of cell migration to be slower than those of inflammatory mediators, with previous publications reporting macrophages to move diffusively at rates of order $10^{-13}$–$10^{-7}~\text{ cm }^2\text{ s }^{-1}$ (Lauffenburger and Kennedy 1983; Sozzani et al. 1991; Owen and Sherratt 1997; Owen et al. 2004). Neutrophils, on the other hand, are expected to move more rapidly owing to their smaller size.

We, here, have the flexibility to tune both the domain size $L^*$ and the timescale $\gamma _c^*$ in our model, to enable us to relate our simulations to some specific inflammatory condition. However, since we are more interested in examining the general case here, we begin by defining a baseline set of dimensionless spatial parameters, which we use in our simulations in Sect. 3.1. We then examine how variations in these parameters impact upon the model’s behaviour in Sect. 3.2. Considering a domain of width $L^*=10~\text{ cm }$, taking $\gamma _c^*=3~\text{ day }^{-1}$ (as in Dunster et al. (2014)), and taking typical (dimensional) rates of diffusion of mediators and macrophages to be $10^{-7}~\text{ cm }^2\text{ s }^{-1}$ and $10^{-10}~\text{ cm }^2\text{ s }^{-1}$, respectively, provides approximate dimensionless estimates of $D_c,D_g=10^{-4}$ and $D_m=10^{-6}$. Since we expect neutrophils to move more quickly than macrophages but slower than mediators, we prescribe $D_n=10^{-5}$. Finally, as an initial point of reference, we assume that chemotaxis parameters are of a similar order to those of diffusion, and hence set $\theta _n=10^{-5}$ and $\theta _m=10^{-6}$; we investigate the effects of variations in the strength of neutrophil and macrophage chemotaxis more thoroughly in Sect. 3.2. These parameter choices are summarised in Table 2.

## Results

In the subsections below we illustrate that the system (3) can exhibit spatially inhomogeneous (patterned) solutions that the ODE model of Dunster et al. (2014) omits. Unless otherwise stated below, we initiate the model using initial conditions that incorporate uniform damage in a circle of radius 0.25 centred at $(x,y)=(0.5,0.5)$. The extent of this damage is determined by the coordinates of the non-trivial steady state exhibited by the ODE that results from eliminating the spatial terms. Outside of this circle, all variables are set to zero, representing healthy tissue. Throughout our analysis, we address the questions of whether the localised damage in the centre of the domain can invade the neighbouring healthy tissue and, if it does so, whether the inflammation is globally resolved in the long term, becomes uniformly persistent (chronic), or whether spatially inhomogeneous solutions can arise.

Numerical solution of PDE systems involving chemotaxis [such as (3)] presents some challenges with regard to numerical stability and the positivity and smoothness of solutions, particularly for choices of parameter values that render chemotactic terms larger than associated diffusion terms. (See, for example, Gerisch et al. 2001; Gerisch and Verwer 2002; Gerisch and Chaplain 2006 for details.) In order to resolve these challenges, we follow the numerical approach of Gerisch et al. (2001), first implementing a spatial discretisation of the 2D domain, and then solving the resulting system of ODEs using a splitting method in which chemotactic terms are timestepped explicitly using a fourth-order Runge–Kutta (RK4) method, and the remaining reaction and diffusion terms are timestepped implicitly using ode15s in Matlab. As described in Gerisch et al. (2001), our numerical method makes use of a van Leer flux limiter (described in detail by Sweby 1984) in order to preserve the positivity of solutions.

In the sections below, we begin with consideration of how the space-free parameters of the corresponding ODE system impact upon the potential to attain spatial patterns. We then examine how the spatial parameters related to cell/mediator diffusion and cell chemotaxis impact upon the spatial patterns observed. Finally, we briefly discuss the sensitivity of our results to our choice of spatial domain and initial conditions.

### Dependence Upon Non-spatial Parameters

**Fig. 3:**
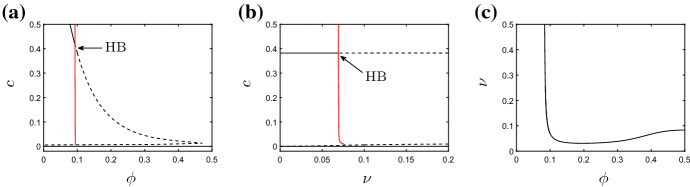
Bifurcation diagrams for the non-spatial system. **a** Bifurcation diagram for $\nu =0.1$ and varying $\phi$. The supercritical Hopf bifurcation (HB) lies at $\phi =\phi _{\mathrm{{HB}}}\simeq 0.09$. **b** Bifurcation diagram for $\phi =0.1$ and varying $\nu$. The supercritical Hopf bifurcation lies at $\nu =\nu _{\mathrm{{HB}}}\simeq 0.07$. In both **a** and **b**, the vertical axis is the pro-inflammatory mediator concentration, *c*. Solid (resp. dashed) black lines indicate stable (*resp.* unstable) fixed points; red lines represent stable periodic orbits. In **c**, we illustrate the location of the Hopf bifurcation in $(\phi ,\nu )$-space; the non-trivial steady state is stable below the curve shown. (All unspecified parameter values are as given in Table 2.) (Colour figure online)

We begin by examining how the parameters that appear in the corresponding ODE model impact upon the spatial patterns exhibited by the PDE model of (3). As described by Dunster et al. (2014), in the absence of spatial terms, the model exhibits a stable steady state in which all variables settle to zero, corresponding to inflammation being resolved and yielding a healthy outcome. This steady state is stable for all biologically feasible choices of parameters. In addition to this (and dependent upon parameter values) the model can exhibit at most one additional stable steady state that corresponds to chronic damage; this steady state can be eliminated via a Hopf bifurcation as key parameters are varied, giving rise to sustained temporal oscillations for some choices of parameters. Dependent on parameter choices, the ODE system is either bistable (with both healthy and chronic steady states permissible), excitable (in which sustained oscillations are present), or monostable (in that attaining the healthy steady state is the guaranteed outcome). We direct the reader to Dunster et al. (2014) for a more thorough review of the above; however, in Fig. 3 we show bifurcation diagrams that illustrate how the stability of the steady states described above depends upon key model parameters. These bifurcation diagrams, which were produced numerically using the continuation software XPP–Auto, act as a starting point for our analysis of the spatially dependent model of (3) below. We focus here upon the effects of varying the rates of neutrophil apoptosis, $\nu$, removal of apoptotic neutrophils by macrophages, $\phi$, and production of anti-inflammatory mediators, $k_g$, as these parameters are identified as amongst the most significant in the analysis of the corresponding ODE model (Dunster et al. 2014) and represent potential therapeutic targets. All other parameters are held fixed at the values given in Table 2 unless otherwise stated.

**Fig. 4:**
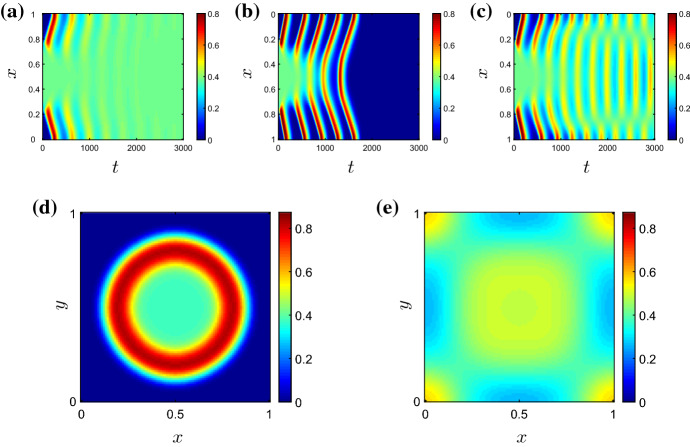
Solutions of (3) for varying $\nu$, and all other parameters as given in Table 2. In **a**–**c**, we plot the pro-inflammatory mediator concentrations on the cross section $y=0$ as a function of time. **a** For $\nu =0.05$ ($\nu <\nu _{\mathrm{{HB}}}$), the damage initially located in the centre of the domain spreads globally, and the system ultimately attains the non-trivial (chronic) homogeneous steady state given by the corresponding ODE model. **b** For $\nu =0.1$ ($\nu \gg \nu _{\mathrm{{HB}}}$), the system ultimately attains a globally resolved configuration, since the chronic steady state in the ODE model is unstable for this choice of $\nu$. **c** For $\nu =0.075$ ($\nu >\nu _{\mathrm{{HB}}}$), the system exhibits temporal oscillations that are inhomogeneous in space. Snapshots of the spatial profile of pro-inflammatory mediator for $\nu =0.075$ are also shown at **d** $t=100$ and **e** $t=2000$ (Colour figure online)

In order to more clearly visualise the temporal dependence of our 2D solutions in the analysis that follows, we often illustrate (in Fig. 4a–c, for example) the temporal evolution of spatial patterns by considering a cross section of the two-dimensional domain at $y=0$ and plotting the corresponding information against time. High (*resp.* low) concentrations of system variables are shown in red (*resp.* blue).

*Varying rates of neutrophil apoptosis* In Fig. 4, we illustrate solutions arising for various choices of $\nu$, with all other parameters fixed at those values gives in Table 2. In the absence of spatial terms, the bifurcation diagram of the corresponding ODE model is as shown in Fig. 3b and exhibits a supercritical Hopf bifurcation at $\nu =\nu _{\mathrm{{HB}}}\simeq 0.07$. In a spatial domain, setting $\nu =0.05$ allows the damage in the centre of the domain to rapidly invade the neighbouring healthy tissue (Fig. 4a). The system quickly attains a configuration that corresponds to uniform damage of magnitude given by the corresponding branch of the bifurcation diagram in Fig. 3b. (As indicated by the corresponding ODE model, the system is bistable in this region of parameter space; the magnitude of the damage imposed in the initial condition acts as a switch between the uniformly healthy or damaged outcomes.) Similar behaviour occurs for all values of $\nu <\nu _{\mathrm{{HB}}}$. For $\nu =0.1$, a choice of $\nu$ significantly greater than $\nu _{\mathrm{{HB}}}$, the non-trivial homogeneous steady state is unstable in the ODE model and, despite damage initially spreading into the neighbouring healthy tissue via a number of pulses emanating radially from the centre of the domain, the system ultimately converges to a uniformly healthy configuration (Fig. 4b). However, for choices of $\nu >\nu _{\mathrm{{HB}}}$ that are relatively close to the Hopf bifucation, we observe that the system supports sustained, spatially inhomogeneous, temporal oscillations (as illustrated for $\nu =0.075$ in Fig. 4c–e). The long-term pattern in this case is one of a ‘spotted checkerboard’ structure, as shown in Fig. 4e, whose amplitude oscillates temporally.

**Fig. 5:**
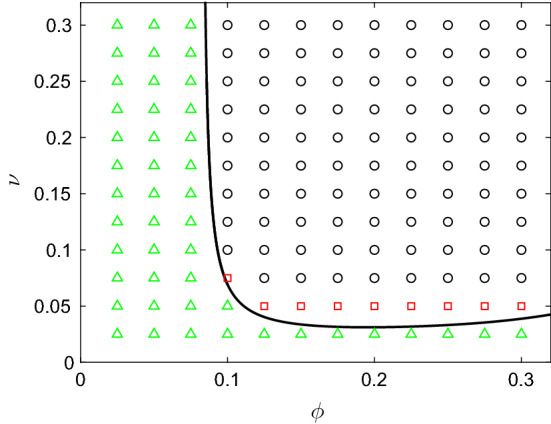
Summary of the types of solutions emitted by (3) for various choices of $\nu$ and $\phi$, and all other parameter values as given in Table 2. Green triangles indicate that the system attains the non-trivial (chronic) homogeneous steady state given by the ODE model; red squares indicate that the model exhibits spatially inhomogeneous temporal oscillations; black circles indicate that the damage is resolved uniformly. The black curve marks the location of the Hopf bifurcation (Colour figure online)

*Varying rates of neutrophil apoptosis and macrophage phagocytosis* In Fig. 5, we summarise where in $(\nu ,\phi )$-space we find each of the broad solution types above, with the remaining parameters fixed at the values given in Table 2. For all choices of $\nu$ and $\phi$ that fall to the left of (or below) the Hopf bifurcation curve (shown in black in Fig. 5), the system converges to a stable homogeneous steady state corresponding to uniform damage (demarked by green triangles in the figure). For the majority of parameter choices that fall significantly to the right (or above) the Hopf bifurcation curve, the system ultimately progresses towards the trivial homogeneous steady state at zero, corresponding to damage being uniformly resolved (as shown by black circles in the figure). We note that in these areas of parameter space, the non-trivial homogeneous steady state is unstable, so uniform chronic damage is not a permissible configuration. However, for suitable choices of $\phi$, there exists a narrow region of parameter space immediately beyond the Hopf bifurcation in which long-term spatially inhomogeneous configurations exist. For the parameter choices represented by red squares in Fig. 5, these configurations display oscillations temporally as well as spatially; however, spatially inhomogeneous steady states can also be permissible for some choices of parameters, as the following section will illustrate. We note that these spatially inhomogeneous configurations do not fall into the classical class of solutions that typically arise through Turing instabilities, given their temporally oscillating nature (in most cases), and that they do not result from changes in the stability of the homogeneous steady states in the corresponding ODE model. These results are akin to similar patterns driven by Hopf bifurcation in other models, such as that of Penner et al. (2012), for example. Below, we examine how variations in the choices of spatial parameters effect the spatially dependent configurations observed here.

**Fig. 6:**
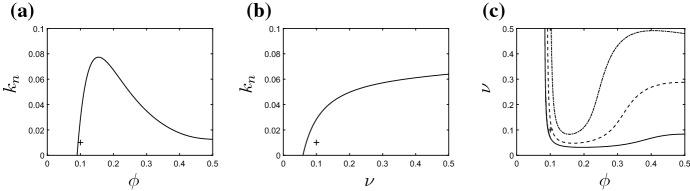
Bifurcation diagrams illustrating the effects of the neutrophil feedback parameter $k_n$ upon locations of Hopf bifurcations as functions of **a** $\phi$ and **b** $\nu$. In **a**, **b**, the non-trivial steady state is stable for parameter combinations above the black curves. In **c** we show the position of the Hopf bifurcation in $(\phi ,\nu )$-space for $k_n=0.01$ (solid line), $k_n=0.04$ (dashed line) and $k_n=0.07$ (dash-dotted line). The non-trivial steady state is stable below the illustrated curves; the areas of parameter space above the curves exhibit potential for inhomogeneous solutions. The cross symbols demark the baseline parameter values of Table 2

*Varying the neutrophil feedback rate* The model of (3) incorporates a positive feedback loop owing to the production of pro-inflammatory mediators by active neutrophils, the associated rate constant being denoted $k_n$. In Fig. 6, we briefly examine the extent to which this positive feedback loop impacts upon potential solutions. As discussed above, the facet of the model that is key in determining the long-term outcome is the position of the Hopf bifurcation. For parameter combinations that lie prior to the Hopf bifurcation, the model is bistable and we have the potential to obtain either healthy or chronic homogeneous steady states dependent upon initial conditions; for parameter choices beyond the Hopf bifurcation, the only permissible solutions are the healthy homogeneous configuration or spatially inhomogeneous outcomes. In Fig. 6, we illustrate how the position of the Hopf bifurcation depends upon our choice of $k_n$. In Fig. 6a, b, we present two-parameter bifurcation diagrams that illustrate how the $\phi$- and $\nu$-coordinates of the Hopf bifurcations evolve as $k_n$ is varied; the non-trivial homogeneous steady state is stable for parameter combinations that lie above the illustrated curves. As $k_n$ is increased from our baseline value of $k_n=0.01$, there is a narrowing window of $\phi$-values (for fixed $\nu$) for which the non-trivial steady state is unstable (Fig. 6a). As such, the enhanced pro-inflammatory mediator production by neutrophils essentially acts to enhance the stability of the non-trivial steady state, hence increasing the potential of attaining a globally chronic state. Holding $\phi$ fixed and varying $k_n$, the position of the Hopf bifurcation is an increasing function of $\nu$, since the enhanced apoptosis of neutrophils acts to counter the pro-inflammatory mediator production by active neutrophils (Fig. 6b). For the parameter values of Table 2, choices of $k_n\gtrsim 0.07$ result in the Hopf bifurcation being eliminated completely. Figure 6c illustrates the curve in $(\phi ,\nu )$-space on which the Hopf bifurcation lies, for various choices of $k_n$. Below the illustrated curves, the model attains a homogeneous outcome (either healthy or chronic); we infer from Fig. 5 that the region of viable inhomogeneous solutions lies immediately above these curves. In general, increases in $k_n$ act to overwhelm spatial inhomogeneities, biassing the system towards the chronic homogeneous outcome.

**Fig. 7:**
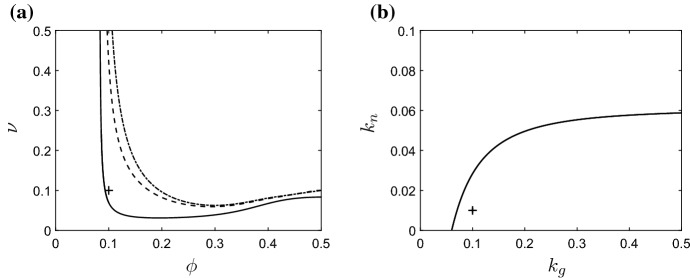
Bifurcation diagrams illustrating the role of the anti-inflammatory mediator. In **a** we show the position of the Hopf bifurcation in $(\phi ,\nu )$-space for $k_g=0.1$ (solid line), $k_g=0.01$ (dashed line) and $k_g=0$ (dash-dotted line). The non-trivial steady state is stable below the illustrated curves; the areas of parameter space above the curves exhibit potential for inhomogeneous solutions. In **b**, we illustrate the location of the Hopf bifurcation in $(k_g,k_n)$-space. The cross symbols demark the baseline parameter values of Table 2

*The role of the anti-inflammatory mediator* The anti-inflammatory mediator, *g*, plays a key role in mediating the inflammatory outcome by both suppressing the recruitment of new neutrophils and enhancing the rate at which existing active neutrophils become apoptotic. We, here, examine how the outcomes described above depend upon the levels of anti-inflammatory mediator in the system, focusing in particular upon the influence of variations in the anti-inflammatory mediator production rate, $k_g$. In Fig. 7, we illustrate how the positions of the Hopf bifurcations depend upon $k_g$. In Fig. 7a, we show how the curve of Hopf bifurcations that separates regions of bistability from potential regions of inhomogeneous outcomes moves in $(\phi ,\nu )$-space as $k_g$ varies. Below the illustrated curves, the system is bistable with both healthy and chronic homogeneous solutions being stable; above the illustrated curves the chronic steady state is unstable, and the only permissible unhealthy solution is one of spatial inhomogeneity. Decreasing $k_g$ has a similar effect to increasing the neutrophil feedback parameter $k_n$ (as described above) in that the chronic steady state becomes stable for a wider range of parameters in $(\phi ,\nu )$-space. This can bias the system towards the chronic homogeneous outcome, in a manner that is dependent upon initial conditions. In Fig. 7b, we plot the Hopf bifurcation position in $(k_g,k_n)$-space, with all other parameters fixed at the values given in Table 2. Both chronic and healthy homogeneous solutions are stable above the illustrated curve; inhomogeneous solutions are permissible below this curve. In the limit $k_g\rightarrow 0$, inhomogeneous solutions are eliminated entirely. Finally, we note that qualitatively similar observations may be made when manipulating other parameters relating to the anti-inflammatory mediator; increases in $\gamma _g$ or $\beta _g$ have the effect of reducing the concentration of the anti-inflammatory mediator or reducing its influence upon neutrophil apoptosis, respectively, both of which result in the Hopf bifurcation curve approaching the dash-dotted line of Fig. 7a (for which $k_g=0$). These results are omitted for brevity.

**Fig. 8:**
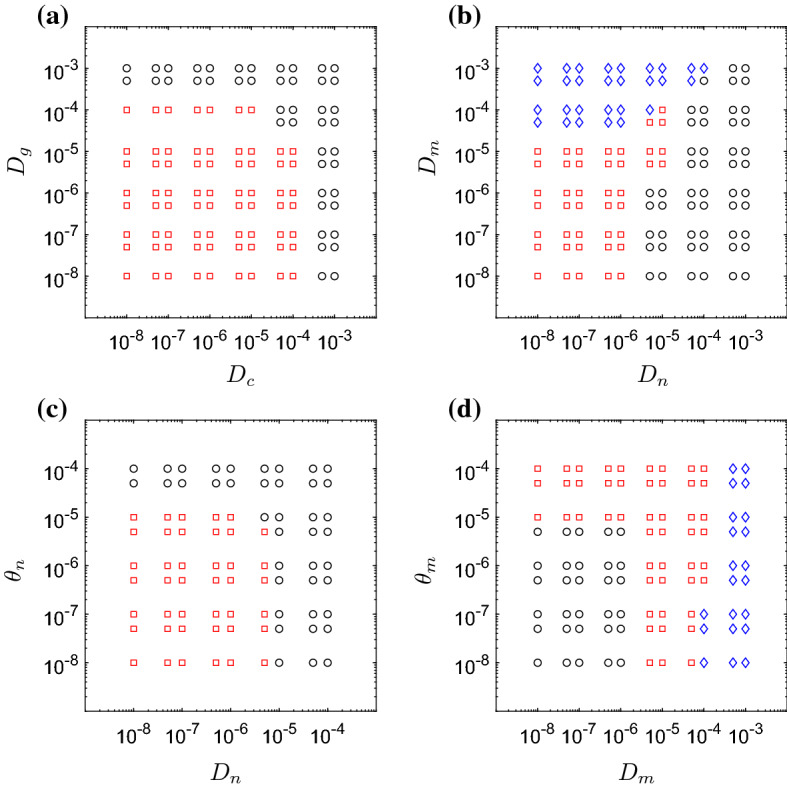
Results obtained for various choices of mediator/cellular diffusion rates ($D_c$, $D_g$, $D_n$ and $D_m$) and cell chemotaxis parameters ($\theta _n$, $\theta _m$). In each panel we vary two of these six parameters, holding all other parameters fixed at the values given in Table 2. Red squares indicate that the model exhibits spatially inhomogeneous oscillations; blue diamonds indicate that the system attains a spatially inhomogeneous steady state; black circles indicate that damage is resolved uniformly (Colour figure online)

### Dependence Upon Spatial Parameters

We here investigate how variations in the rates of cell/mediator diffusion ($D_n$, $D_m$, $D_c$, $D_g$) and cell chemotaxis ($\theta _n$, $\theta _m$) effect the permissibility of spatial patterns. Starting with the baseline set of parameters given in Table 2, we vary each of these six spatial parameters individually. Given that we hold the non-spatial parameters fixed at the choices given in Table 2, values for which spatial patterns have been identified as possible, we know that the only potential outcomes here are (i) a uniformly resolved configuration in which all variables reach the steady state at zero, or (ii) a configuration that exhibits spatial inhomogeneity. Since our parameter choices lie to the right of the Hopf bifurcation in Fig. 3, we do not expect to attain a configuration that represents uniform damage, in particular.

In Fig. 8, we illustrate the manner in which the spatial homogeneity of solutions is dependent upon these spatial parameters. In each panel, we vary two of the six spatial parameters, holding the other four at the values of Table 2. As Fig. 8 illustrates, there are two broad routes through which spatial patterns can be suppressed. Firstly, parameter sets for which pro-inflammatory mediator diffusion ($D_c$) or neutrophil motility ($D_n$, $\theta _n$) are high initially result in damage spreading across the domain, until the domain becomes globally inflamed. This triggers a global inflammatory response that ultimately yields uniform resolution (as demarked by black circles in Fig. 8), given that a steady-state configuration corresponding to uniform damage is not permissible here. On the contrary, high rates of diffusion of anti-inflammatory mediator ($D_g$) result in high levels of this mediator across the domain, thus suppressing the recruitment of neutrophils globally, and limiting the initial spread of damage. The system once again attains the trivial (healthy) steady state emitted by the corresponding ODE model. In general, spatially inhomogeneous solutions are attainable when spatial parameters take low to moderate (physiological) values. It is interesting to note that the model supports both spatially inhomogeneous steady states and spatially inhomogeneous solutions that oscillate temporally (as demarked by blue diamonds and red squares in Fig. 8, respectively). The rate of macrophage diffusion ($D_m$) seems to play a key role in acting as a switch between these two broad outcomes, as Fig. 8b illustrates.

As Fig. 8c, d illustrates, the extent to which neutrophil and macrophage migration are directed (i.e. chemotaxis-dominated) or undirected (i.e. diffusion-dominated) has a relatively weak influence upon the spatial homogeneity of solutions in general. For a combination of parameters that results in spatially inhomogeneous oscillations, for example, we can move the system towards uniform resolution of damage by either increasing neutrophil motility (through $D_n$ or $\theta _n$) or decreasing macrophage motility (through $D_m$ or $\theta _m$). We note that, in this model, the weak impact of the chemotaxis terms is partially attributable to the fact that neutrophils and macrophages can also be recruited directly to the damage site from the underlying tissue, thus rendering directed migration of cells across the domain less important for resolving localised inflammation. It is noteworthy that for small rates of neutrophil diffusion ($D_n$), the netrophil chemotaxis parameter, $\theta _n$, can act as a switch between healthy and chronic outcomes.

**Fig. 9:**
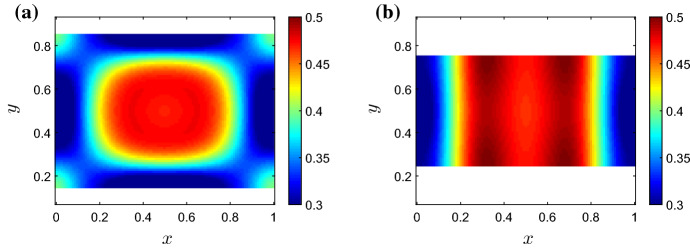
Snapshots (at time $t=2000$) of temporally oscillating, spatially inhomogeneous configurations attained on rectangular domains comprised of $x\in [0,1]$ and **a** $y\in [0.15,0.85]$, and **b** $y\in [0.25,0.75]$, for $\nu =0.075$ and all other parameter values as given in Table 2. Moving from a square domain (Fig. 4e) to a narrowing rectangular domain can drive the system from spotted to striped patterns (Colour figure online)

**Fig. 10:**
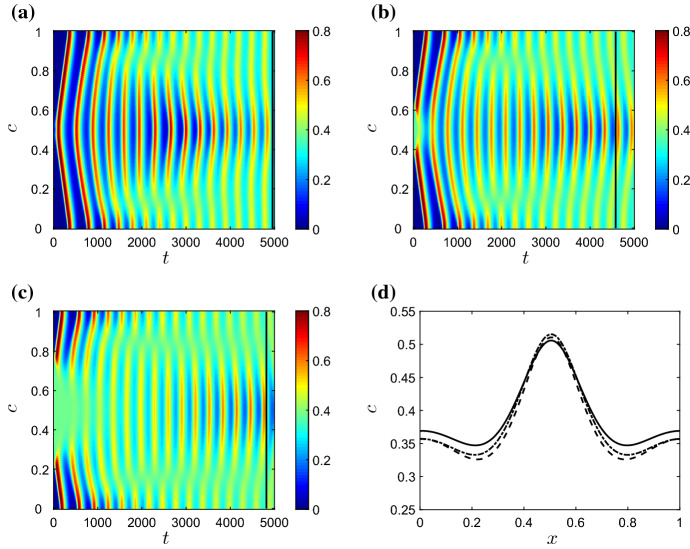
The effect of changing the radius of the initial damage upon pro-inflammatory mediator concentrations, *c*, for $\nu =0.075$ and $\phi =0.1$. In **a**–**c** we plot the concentrations of *c* on the cross section $y=0$ as functions of time, with initially damaged areas of radius **a** 0.01, **b** 0.1, and **c** 0.25. In **d** we plot the mediator distributions at the times demarked by the black lines in **a**–**c**, with solid, dashed and dash-dotted lines relating to panels **a**, **b** and **c**, respectively. The long-term profiles are qualitatively similar for all three configurations (Colour figure online)

### Domain Effects and Dependence Upon Initial Conditions

We, here, briefly assess the extent to which the patterns identified above are sensitive to our choice of domain. Numerical simulations conducted on a number of rectangular domains of varying aspect ratio, with $\nu =0.075$ ($\nu >\nu _{\mathrm{{HB}}}$) and all other parameters as in Table 2, reveal that the regions of parameter space in which we attain uniformly resolved, uniformly damaged, or spatially inhomogeneous configurations are exactly as illustrated in Fig. 5. (Results omitted for brevity.) However, changes in the shape of the domain can impact on the resultant patterns themselves. In Fig. 9, we illustrate snapshots of two solutions attained on domains with *y* restricted to the intervals (a) [0.15, 0.85] or (b) [0.25, 0.75] (with $x\in [0,1]$ as previously). Comparing these results to those attained on the square domain (Fig. 4e), we observe that moving to a domain with a higher aspect ratio can predispose the system to striped patterns such as that shown in Fig. 9b. Additional simulations also reveal that replacing periodic boundary conditions by Neumann boundary conditions on all boundaries has negligible impact upon permissible patterns. (Once again, results are omitted for brevity.)

In Fig. 10, we examine the sensitivity of patterns to the size of the area of damage imposed via the initial conditions described above. In the figure, we set $\nu =0.075$ and $\phi =0.1$ (a parameter choice for which we have demonstrated, in Fig. 5, that temporally oscillating patterns are permissible), fix all other parameters at the values used given in Table 2, and examine results for various choices of the radius of the initially damaged area. As the figure shows, while variations in the radius of the initial damage give rise to some differences in the short term, long-term patterns are largely insensitive to the size of the initially damaged area. Starting with uniform damage of course results in spatially homogeneous results (not shown), but since this configuration is unstable in the ODE model, introducing even a small amount of healthy tissue is sufficient to allow the PDE system to diverge from the unstable homogeneous configuration and instead attain stable, spatially inhomogeneous periodic orbits. Similar simulations (not plotted here for brevity) reveal that the model is also largely insensitive to changes in the magnitude of the initial damage. For example, on making all the initial conditions used above ten times larger or smaller, we attain qualitatively equivalent spatially inhomogeneous solutions in the long term, despite some small variations in initial behaviour.

## Discussion

In this work we have extended an existing homogeneous model of the acute inflammatory response to include the spatial effects of cell motility and mediator diffusion. In contrast to many previous models of the inflammatory response that take the approach of considering only generic populations of white blood cells (Lauffenburger and Keller 1979; Lauffenburger and Kennedy 1981, 1983; Kumar et al. 2004; Reynolds et al. 2006), our model captures the key interactions between the distinct cell populations thought to play a fundamental role in determining the inflammatory outcome, be it homogeneous resolution or progression to a chronic, self-perpetuating state. Through the inclusion of spatial descriptions of immune cells and inflammatory mediators, our extended model enables us to elucidate the influence of cell migration and mediator diffusion upon the spatial spread of inflammatory damage and its ability to resolve within a spatial setting.

We have analysed our model both analytically, via a bifurcation analysis of the corresponding homogeneous model, and through extensive numerical simulation of our spatially dependent extension. Guided by an existing focus in the literature upon targeting the phagocytosing ability of macrophages ($\phi$) in the hunt for new therapeutic strategies (see e.g. Porcheray et al. 2005; Serhan 2017), together with the fact that the analysis of Dunster et al. (2014) identified the neutrophil apoptosis rate ($\nu$) as a key parameter in determining the nature of long-term outcomes, our analysis initially focused upon how variations in these two parameters can facilitate or inhibit spatially inhomogeneous outcomes. While the ODE model shows that variations in these two parameters that result in crossing the Hopf bifurcation curve in Fig. 3c act as a switch from bistability to monostability (i.e. guaranteed resolution of inflammation), simulations of our spatially dependent model illustrate (in Fig. 5) that crossing the same Hopf bifurcations can move the model into an area of parameter space for which persistent spatially inhomogeneous solutions exist—both steady-state solutions and solutions that oscillate temporally. These chronically inflamed solutions lie in an area of parameter space for which the ODE model predicts full resolution of damage. For the majority of parameters studied, our spatially inhomogeneous solutions comprise disparate areas of damage whose severity oscillates temporally; while one area of damage may be resolving, in that pro-inflammatory mediator levels are reducing, other areas of damage are worsening due to the feedback from both active and apoptotic neutrophil populations. Oftentimes, monitoring the pro-inflammatory mediator concentrations alone would seem to indicate resolution, yet responses from the other components of the system yield further flares of damage in due course. In this sense, the temporal patterns that we observe are reminiscent of inflammatory conditions that have relapsing–remitting characteristics, such as Crohn’s disease or rheumatoid arthritis (Tibble et al. 2000; Firestein 2003).

Having established the areas of $(\phi ,\nu )$-space that exhibit spatially inhomogeneous solutions, we then examined the extent to which the balance between the pro-inflammatory feedback from active neutrophils (with rate $k_n$) and the counteracting role of the anti-inflammatory mediator (*g*) can have a key influence upon biassing the system towards globally inflamed (homogeneous) or spatially inhomogenous solutions (as illustrated in Figs. 6 and 7). For large choices of $k_n$ or small concentrations of anti-inflammatory mediator (e.g. in the limit $k_g\rightarrow 0$), spatial patterns can be eliminated and the results of the corresponding homogeneous model are recovered. While some drugs in current usage (such as methotrexate, sulphasalazine and FK506) do act to mitigate against inflammation by triggering the synthesis of anti-inflammatory mediators (Gilroy et al. 2004; Haskó and Cronstein 2004), manipulation of anti-inflammatory mediators remains an active area of focus in the hunt for new therapeutic targets (Henson 2005; Barnig et al. 2018; Bäck et al. 2019). Our results indicate that, while increasing the concentrations of anti-inflammatory mediators can move the homogeneous system from a bistable regime (in which chronic outcomes are permissible) to a healthy state, intermediate levels of anti-inflammatory mediators can yield spatially inhomogeneous, non-resolving outcomes.

For spatially independent parameter values that allow the model to emit spatially inhomogeneous solutions, we have explored (in Fig. 8) the extent to which variations in spatial parameters can influence the solutions obtained. For rapidly spreading mediators ($D_c$ and $D_g$ large), the initial damage rapidly spreads to fill the entire domain, triggering a global response that results in a homogeneous, healthy outcome. Similarly, large choices of the neutrophil motility parameters ($D_n$ and $\theta _n$) or small choices of macrophage motility parameters ($D_m$ and $\theta _m$) result in a rapid spread of damage driven by the apoptosis and eventual necrosis of neutrophils, the associated positive feedback in pro-inflammatory mediator concentrations once again triggering a global response that restores the healthy state. For small to moderate choices of the neutrophil diffusion parameter $D_n$, our simulations reveal that strong neutrophil chemotaxis ($\theta _n$ large) can drive resolution of inflammation, while weaker neutrophil chemotaxis can result in a persisting spatially inhomogeneous outcome (Fig. 8c). The role of neutrophil migration in many different inflammation-related pathologies is of increasing interest (Brubaker et al. 2013; Cecchi et al. 2018); there is strong evidence in the biological literature that a reduction in the rate of neutrophil chemotaxis occurs under trauma and ageing and results in an otherwise healthy inflammatory response being pushed into a persistent inflammatory response (Sapey et al. 2014). Indeed, neutrophil migration is now thought to be an attractive therapeutic target for diseases such as chronic obstructive pulmonary disease, a chronic lung disease characterised by aberrant neutrophil migration (Sapey et al. 2011; Jasper et al. 2019).

It is pertinent to remark briefly that, while the model presented here incorporates a reasonably thorough catalogue of biological interactions, this comes at the expense of restricting the use of some key mathematical analyses that would commonly be used in analysing pattern-forming systems. For example, our model does not readily lend itself to travelling wave analysis due to the complexities inherent in the numerous nonlinear reaction and chemotactic terms, and does not allow spatially interesting configurations to be determined analytically; we are therefore restricted to the use of robust numerical schemes in order to identify the model’s spatially dependent solutions. This is in contrast to a broad range of existing models, the construction of which can often omit key biological feedbacks in order to facilitate greater analytical progress. Furthermore, we note that, while we regard the model described here to include the majority of crucial biological interactions at play in a typical inflammatory response, there is certainly scope for inclusion of further, more detailed mechanisms. To do so within the confines of a PDE-based model would likely result in a model that is not easily penetrable via mathematical analysis. This potentially motivates the need for a shift to an alternative modelling paradigm, via which the full remit of biological interactions can be easily incorporated. The use of ‘agent-based’ or ‘cellular automata’ models has become increasingly popular in this regard in recent years, and has shown great success in modelling a range of intricate biological systems, with examples including tumour growth (Gerlee and Anderson 2007; Figueredo et al. 2014), bone remodelling (Tovar et al. 2004), immune responses in the gut (Verma et al. 2019), within-host progression of sexually transmitted infections (Nelson et al. 2014) and many more. Development of advanced cellular automata models of the acute inflammatory response, and comparison of results with PDE-based models such as that described here, remains a key target for ongoing work in this area.

In summary, we have extended an existing spatially homogeneous description of the inflammatory response to include key spatial effects. Our model illustrates that the inflammatory outcome exhibits strong dependence upon the interplay between apoptosing neutrophils, phagocytosing macrophages and anti-inflammatory mediators. For parameter choices close to the switch between bistability and guaranteed resolution in the homogeneous model, our spatial extension exhibits inhomogeneous outcomes. In line with recent experimental data, we find that aberrant neutrophil migration can play a role in the failure of inflammation to resolve. All of the above mechanisms remain key targets in the ongoing search for new therapeutic interventions.
